# Determination of Vitamin A Total Body Stores in Children from Dried Serum Spots: Application in a Low- and Middle-Income Country Community Setting

**DOI:** 10.1093/jn/nxaa446

**Published:** 2021-03-23

**Authors:** Anthony Oxley, Reina Engle-Stone, Jody C Miller, M F Dolly Reario, Ame Stormer, Mario V Capanzana, Carl V D Cabanilla, Marjorie J Haskell, Georg Lietz

**Affiliations:** Human Nutrition Research Centre, Population Health Sciences Institute, Newcastle University, Newcastle upon Tyne, United Kingdom; Institute for Global Nutrition, Department of Nutrition, University of California, Davis, CA, USA; Institute for Global Nutrition, Department of Nutrition, University of California, Davis, CA, USA; Helen Keller International, Malate, Manila, Philippines; Helen Keller International, Malate, Manila, Philippines; Food and Nutrition Research Institute, Department of Science and Technology, Bicutan, Taguig City, Philippines; Food and Nutrition Research Institute, Department of Science and Technology, Bicutan, Taguig City, Philippines; Institute for Global Nutrition, Department of Nutrition, University of California, Davis, CA, USA; Human Nutrition Research Centre, Population Health Sciences Institute, Newcastle University, Newcastle upon Tyne, United Kingdom

**Keywords:** vitamin A status, total body stores, retinol isotope dilution, dried serum spots, dried blood spots, stable isotopes, liquid chromatography tandem mass spectrometry, children, Philippines

## Abstract

**Background:**

The retinol isotope dilution (RID) method has been used to evaluate vitamin A (VA) status in healthy adults and children in low- and middle-income countries (LMIC) and to assess the efficacy of various VA interventions.

**Objective:**

The study was designed to examine whether dried serum spots (DSS) can be applied to RID when conducting VA total body store (TBS) assessments in community settings.

**Methods:**

Four days after an oral dose of 0.4 mg [^13^C_10_]retinyl acetate was administered to Filipino children (12–18 mo), a single blood draw was divided to isolate both serum and plasma. Serum (40 μL) was spotted and dried on Whatman 903 cards and shipped at ambient temperature whereas liquid plasma (LP) was frozen at –80°C and shipped on dry ice. The VA tracer to tracee ratio from DSS and LP was quantified by LC-MS/MS. Comparisons between DSS and LP paired samples (*n* = 72) were made for [^13^C_10_]retinol specific activity (SAp) by Pearson's correlation and for VA TBS by Bland–Altman analysis.

**Results:**

The sum of 3 coextracted DSS were required to consistently detect [^13^C_10_]retinol above the LC-MS/MS limit of quantitation (LOQ). [^13^C_10_]retinol SAp from DSS was highly correlated with SAp from LP (*r* = 0.945; *P* < 0.01). A comparison of methods for TBS determination using Bland–Altman analysis indicated agreement with an intraindividual difference of 24.7 μmol (4.6%). Mean total liver reserve (TLR) values from DSS and LP were 1.7 μmol/g (± 0.6 SD) and 1.6 μmol/g (± 0.6 SD), respectively.

**Conclusions:**

VA TBS can be determined from DSS thereby reducing the logistics and cost of maintaining a cold chain by shipping samples at ambient temperature and, thus, making the RID technique more feasible in LMIC community settings. This trial was registered at https://clinicaltrials.gov as NCT03030339.

## Introduction

Vitamin A deficiency (VAD) is a public health issue in low- and middle-income countries (LMIC), particularly impacting young children and pregnant women in sub-Saharan Africa and South Asia (1). Due to the role of vitamin A (VA) in mucosal epithelial integrity and immune function, VAD significantly increases the severity of illness to common childhood diseases such as measles and diarrhea (2, 3). As such, VA supplementation interventions are strongly recommended for children aged 6–59 mo in order to reduce morbidity and mortality in VAD populations (4). The efficacy of these interventions has been evaluated in both clinical and community settings in LMIC using the retinol isotope dilution (RID) technique (5). The RID method is also useful in assessing VA total body stores (TBS) in studies that observe overlapping VA interventions, since increased liver VA stores above the Biomarkers of Nutrition for Development (BOND) cut-off for hypervitaminosis A of 1 μmol/g liver have previously been reported (6–8).

The main restrictions on performing population-based VA status assessments in community settings are the preparation of serum/plasma from venous blood and the maintenance of a cold chain until sample analysis (8). Dried blood spots (DBS) and dried serum spots (DSS) were first applied to VA surveys in the 1990s to reduce the logistical demands and associated costs of transporting frozen serum to specialized analytical laboratories (9, 10). Since then, primarily due to the ease of finger- and heel-prick blood collection in the field, research has mainly focused on further developing DBS methodologies to measure retinol-binding protein (RBP) by ELISA (11–13) or retinol by HPLC (14–18) and LC-MS/MS (19). Although serum retinol is the most commonly used indicator of VAD, retinol complexed with RBP is under strict homeostatic regulation and, therefore, serum concentrations only correlate with liver VA stores when stores are very low (8). Currently, the RID technique represents the most quantitative estimate of TBS and can determine both VAD and VA status in the range of marginal to high VA stores (5).

Although DBS have been successfully applied in the quantitation of micromolar concentrations of retinol and RBP, RID challenges the MS limit of quantitation (LOQ) of VA stable isotope tracers from blood microsampling devices. As well as limited sample volume, tracer detection above the LOQ is compounded by the physiological dose of tracer that is required to prevent perturbation of endogenous VA body pools. As circulating retinol is confined to the serum/plasma fraction of blood, DSS were employed over DBS in this study to maximize tracer recovery for detection by LC-MS/MS. Therefore, the aim of this study was to assess the feasibility of DSS for TBS determination using the RID technique.

## Methods

### Materials

Whatman 903 protein saver cards (EU) and foil barrier resealable bags were purchased from Scientific Laboratory Supplies Ltd. The stable isotope retinyl acetate (8,9,10,11,12,13,14,15,19,20–^13^C_10_), of 98.8% *all-trans* isomeric purity and >99% ^13^C isotopic enrichment, was custom-synthesized by BuChem BV and conformed to Food Chemicals Codex 4th Edition testing. Acetic acid, formic acid, ethanol, methanol, and acetonitrile were purchased from Fisher Scientific and were certified for HPLC gradient analysis. Retinol and retinyl acetate were obtained from Sigma-Aldrich and were of >99% *all-trans* purity.

### Stable isotopes

[^13^C_10_]Retinyl acetate crystals were dissolved in sunflower oil, at a concentration of 2.5 mg/mL, by heating to 50°C in a water bath and sonicating for 30 min. Complete dissolution of crystals was checked under a light microscope (200× magnification) with the absolute concentration of doses confirmed spectrophotometrically in hexane at λ_max_ 325nm using an E^1%^_1cm_ of 1590 (20). Stable isotope oil preparations were aliquoted into amber glass vials and stored at −20°C for a period of ≤6 mo before dosing.

### Subjects and experimental design

Filipino children (*n* = 123), aged 12 to 18 mo, were enrolled under the multicenter Global Vitamin A Safety Assessment (GloVitAS) project as previously described (21, 22). The GloVitAS Philippines study protocol was approved by both the Research Ethics Board of the University of the Philippines, Manila (IRB ID: UPMREB 2016–282-01) and the Institutional Review Board at UC, Davis (IRB ID: 903,681–2). The study was registered at clinicaltrials.gov as NCT03030339. The experimental design utilized a “super-child” approach (21, 22) where all children received a 400 μg (1.17 μmol) [^13^C_10_]retinyl acetate oral dose, via direct displacement pipette, on day 0. A blood sample was drawn from all children at 4 d postdose, with an additional blood sample taken at a randomly assigned time point (6 h, 9 h, 12 h, 1 d, 2 d, 7 d, 11 d, 16 d, 22 d, or 28 d). However, only the 4 d blood sample, and not the additional time point sample, was the focus of this DSS methodology for determining VA TBS.

Based on a dietary screening tool of VA intake, children were classified into 3 distinct groups: Group 1 = high intake (>600 μg retinol/d) and vitamin A supplement (VAS) in the past 30 d (*n* = 47); Group 2 = high intake and VAS in the past 3–6 mo (*n* = 39); and Group 3 = low/adequate intake (200–500 μg retinol/d) and VAS in the past 3–6 mo (*n* = 37). After screening, a more detailed dietary VA intake assessment was conducted over the 28-d study period, with a mean of four 24 h dietary recalls per child and one 12 h observed weighed record with 12 h recall in addition to a 30 d questionnaire to capture VA-containing supplement use; methods of the National Cancer Institute were used to analyze the combined dietary data (23, 24).

### Sample collection and transport

Four days after stable isotope administration, a 6 mL venous blood sample was collected and divided equally between serum and K_2_-EDTA plasma BD Vacutainer tubes (Becton Dickinson). After blood was allowed to clot in the serum tubes for 30 min at room temperature, both plasma and serum tubes were centrifuged at 2000 × *g* for 15 min at room temperature. For serum, 40 μL was applied to each of the 5 spots on a Whatman 903 card and allowed to air-dry in the dark for 3 h. Cards were then placed in foil pouches with desiccant and stored at −20°C until shipment to Newcastle at ambient temperature (shipping time was ∼48 h). Aliquots of frozen serum were also shipped on dry ice to the VitMin Lab (Willstaett, Germany) where they were analyzed for concentrations of RBP, C-reactive protein (CRP), α(1)-acid glycoprotein (AGP), ferritin, and soluble transferrin receptor (sTfR) by sandwich ELISA (25). For plasma, aliquots were kept frozen at −80°C until shipment to Newcastle University on dry ice. On arrival in Newcastle, both DSS cards and plasma aliquots were stored at −80°C until analysis. A total of 114 frozen plasma and 72 DSS samples, from the 4 d super-child sampling time point, were received at Newcastle. Therefore, 72 within-subject comparisons of paired plasma and DSS samples were used for TBS determinations.

### Extraction from plasma

Frozen plasma was extracted by the method of Aebischer et al. (26) with modification. Briefly, after thawing, 200 μL of plasma was diluted with 200 μL of deionized H_2_0 and denatured with 400 μL of ethanol containing 100 pmol of retinyl acetate as the internal standard (IS). Retinoids were extracted twice with 2 mL of hexane and the combined supernatants dried under a stream of oxygen-free nitrogen (OFN). The dried residue was reconstituted firstly, in 25 μL of ethanol, and secondly, in 80 μL of acetonitrile/H_2_0 (60:40, v/v).

### Extraction from DSS

DSS were extracted according to the method of Huang et al. (18). The whole 13 mm diameter paper disc, corresponding to 40 μL of dried serum, was excised from the card and cut in half before placing in a 2 mL microcentrifuge tube; 20 pmol of retinyl acetate in 40 μL of ethanol was then added as the IS. Discs were extracted by the addition of 1 mL of methanol, containing 2 mM acetic acid, and gentle agitation on a vortex mixer for 10 min. This procedure was repeated and the combined methanol extracts evaporated to dryness under OFN. Residues were resuspended as described for serum extracts. Where 3 DSS (120 μL of serum) were coextracted in the same tube for analysis, the methanol extraction step was repeated a further time. The number of DSS required was estimated from the [^13^C_10_]retinol peak height signal in single DSS samples <LOQ.

### LC-MS/MS analysis

LC-MS/MS analysis was performed on a Shimadzu Prominence HPLC coupled to an API4000 triple quadrupole mass spectrometer (AB Sciex). Liquid chromatographic conditions were adapted from Kane & Napoli (20) utilizing a Supelcosil ABZ PLUS column (Supelco, 2.1 × 100 mm, 3 μm) and a 4 × 2 mm SecurityGuard (Phenomenex) C_18_ cartridge maintained at 25°C. Mobile phases consisted of A: 0.1% (v/v) formic acid and B: 0.1% (v/v) formic acid in acetonitrile. A reverse-phase gradient elution, at a flow rate of 400 μL/min, was employed: 60% B to 95% B from 0.0 to 6.0 min; 100% B from 6.1 to 13.0 min; 60% B from 13.0 to 14.0 min; and then 60% B from 14.0 to 17.0 min to re-equilibrate the column; 50 μL of sample extract was injected onto the HPLC system. For MS/MS analysis, atmospheric pressure chemical ionization (APCI) in positive ion mode was used under the following optimized ion source conditions: CUR 20 psig; GS1 40 psig; GS2 40 psig; CAD 6 psig; TEM 300°C; NC 5 μA. Analyte mass transitions were *m/z* 269→93 for [^12^C]retinol and [^12^C]retinyl acetate, and *m/z* 279→100 for [^13^C_10_]retinol (27) with selected reaction monitoring (SRM) parameters of: EP 10 V, DP 61 V, CE 47 eV, CXP 6 V. Both [^12^C]retinol and [^13^C_10_]retinol sample concentrations were quantified against a [^12^C]retinol/IS peak ratio calibration curve using Analyst software (AB Sciex). The National Institute of Standards and Technology (NIST) standard reference material (SRM) 968e (fat-soluble vitamins in frozen human serum) was used as a quality control (QC) for plasma retinol concentrations.

### Calculations and statistical analysis

To present descriptive information on dietary VA intake, dietary recall data were processed as described previously (22), and mean VA intake was calculated for each child from available days of data. TBS (μmol) was determined in both plasma and DSS samples at 4 d using the RID equation: TBS = Fa × S × (1/SAp); where Fa is the fraction of administered VA isotope dose that is absorbed and retained in storage pools and S is the ratio of retinol SAp to that in stores after equilibration (28), where a combined FaS factor of 2.44 was used for the Filipino cohort under study (22). Specific activity of the retinol stable isotope tracer concentration (μmol/L) in plasma or serum (SAp) was calculated as: [tracer/(tracer + tracee)]/dose (μmol) (28). Total liver VA reserves (TLR; μmol/g) were estimated by: [(TBS × 0.8)/(body weight  × 0.03)] (29). A Pearson's correlation coefficient (2-tailed) with significance *P* value was calculated using IBM SPSS version 24. Bland–Altman analysis and the linear correlation plot was performed using GraphPad Prism version 8.

## Results

### Subject characteristics

As aforementioned, 72 Filipino infants out of the recruited 123 were used for the TBS method comparison and were of 14 mo mean (11–18 mo) age and 8.9 kg mean (6.7–12.7 kg) weight (**Table 1**). Dietary VA intake estimated during the study period varied greatly but was adequate to high in general (**Table 2**). Mean plasma retinol and RBP concentrations were 1.1 μmol/L and 1.2 μmol/L, respectively. Four individuals displayed retinol <0.7 μmol/L suggesting subclinical VAD (30), however, all 4 cases also had elevated acute phase proteins which indicates circulating retinol was lowered by infection (31). Regarding acute-phase protein markers of inflammation, 13 children had CRP concentrations >5.0 mg/L, 18 children had AGP concentrations >1.0 g/L, whereas 7 children possessed both CRP and AGP concentrations above the clinical cut-offs. The individual with the highest CRP concentration of 95 mg/L also possessed the lowest circulating retinol concentration of 0.6 μmol/L.

**TABLE 1:** Anthropometric and biochemical characteristics of Filipino children measured at 4 d postdose^1^

Characteristic	Value
Males, *n*	39
Females, *n*	33
Age, month	14.1 ± 1.7
Weight, kg	8.9 ± 1.1
Length, cm	73.9 ± 3.4
BMI, kg/m^2^	16.3 ± 1.2
Serum ferritin, μg/L	31.8 ± 25.7
Serum sTfR, mg/L	8.0 ± 2.1
Serum CRP, mg/L	3.7 ± 11.8
Serum AGP, g/L	0.8 ± 0.6

1 Values are means ± SD for participating children (*n* = 72). AGP, α-1-acid glycoprotein; CRP, C-reactive protein; sTfR, soluble transferrin receptor.

**TABLE 2:** Measures of VA intake and status in Filipino children measured at 4 d postdose^1^

VA measure	Value
Dietary VA intake,^2^ μg RAE/d	1020 ± 708
Plasma retinol, μmol/L	1.1 ± 0.3
Serum RBP, μmol/L	1.2 ± 0.3
Plasma TBS, μmol	530 ± 188
DSS TBS, μmol	555 ± 191
Plasma TLR, μmol/g	1.6 ± 0.6
DSS TLR, μmol/g	1.7 ± 0.6

1 Values are means ± SD for participating children (*n* = 72). DSS, dried serum spot; RAE, retinol activity equivalents; RBP, retinol-binding protein; TBS, total body stores; TLR, total liver reserves; VA, vitamin A.

2 Usual dietary VA intake was estimated based on the combined dietary data collected in the four 24 h recalls, a 12 h in-home observation followed by a 12 h recall, and a 30 d questionnaire.

### LC-MS/MS analysis of DSS

**FIGURE 1:**
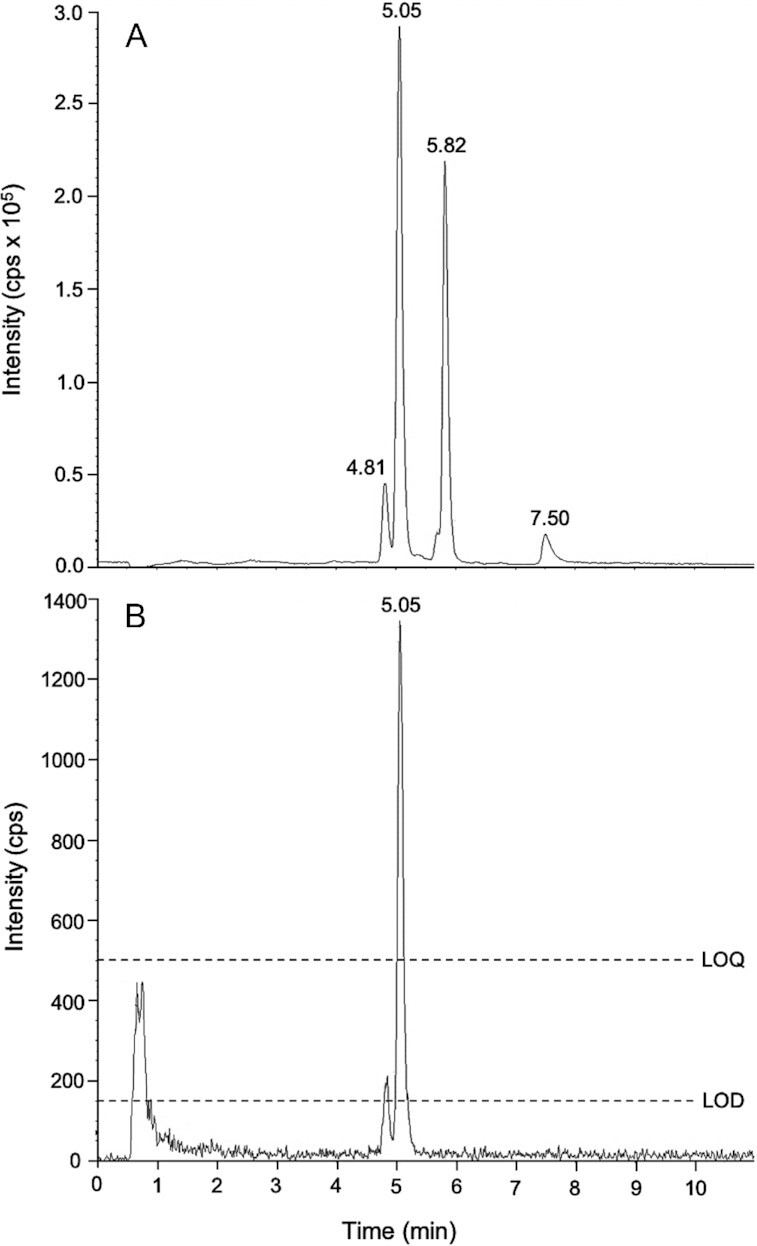
LC-MS/MS chromatogram of a typical dried serum spot (DSS) extract sampled 4 d after oral ingestion of 0.4 mg [^13^C_10_]retinyl acetate. Selected reaction monitoring (SRM) of *m/z* 269→93 (A) shows the separation of endogenous [^12^C]retinol (5.05 min) and the [^12^C]retinyl acetate internal standard (5.82 min). The [^13^C_10_]retinol peak at *m/z* 279→100 (B), from ingested [^13^C_10_]retinyl acetate, sufficiently exceeds both the limit of detection (LOD) and the limit of quantitation (LOQ). Unconfirmed peaks within the *m/z* 269→93 channel were apparent at 4.81 min and 7.50 min. Ion signal intensity is defined as counts per second (cps).

The success rate of [^13^C_10_]retinol tracer detection above the LOQ for single DSS (*n* = 72) analyzed by LC-MS/MS was 79%. However, this rate increased to 100% when 3 DSS were coextracted and employed for analysis. The background noise level within the *m/z* 279→100 transition, and across the retinol elution window, displayed a mean peak height of ∼50 cps (**Figure 1**). This yielded an LOD of 150 cps and LOQ of 500 cps for [^13^C_10_]retinol which corresponded to 6 fmol and 19 fmol on-column, respectively. Optimization of the resuspension solvent composition resulted in 50% of the extract being injected which enhanced analyte detection without compromising peak separation. Liquid chromatographic conditions provided good baseline resolution between the [^12^C]retinol tracee and retinyl acetate IS within the shared *m/z* 269→93 transition. Using the plasma extraction protocol, the accuracy of retinol measurements were in good agreement with 3 retinol levels of NIST certified reference standards: Level 1 = 1.19 μmol/L certified, 1.21 μmol/L obtained; Level 2 = 1.68 μmol/L certified, 1.70 μmol/L obtained; Level 3 = 2.26 μmol/L certified, 2.10 μmol/L obtained. The intra- and interday retinol measurement precision CV from an extracted pooled plasma QC was 2.3% and 4.4%, respectively.

### TBS determination

**FIGURE 2:**
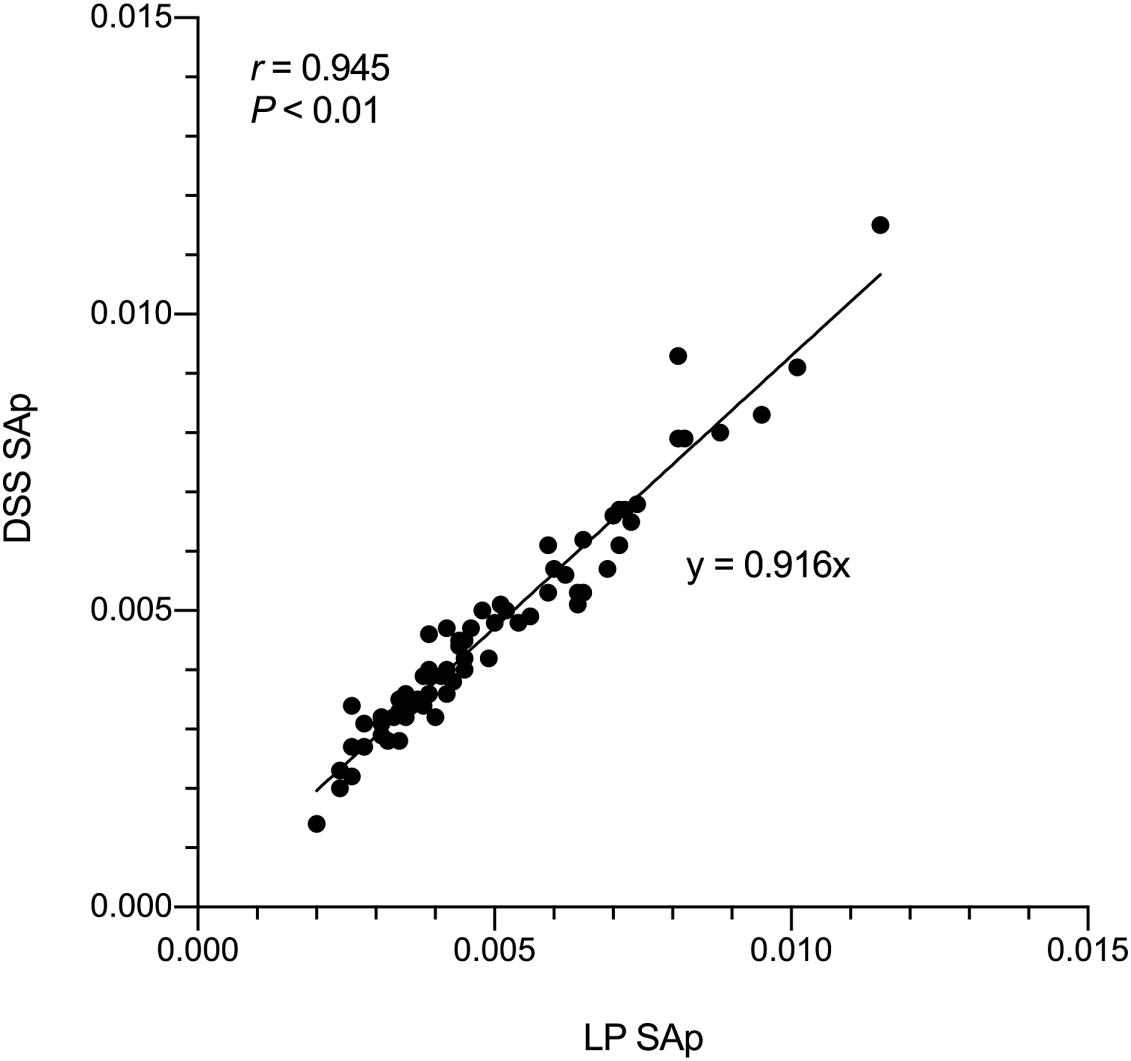
Linear correlation between the specific activity (SAp) of [^13^C_10_]retinol from dried serum spots (DSS) and liquid plasma (LP) paired samples (*n* = 72) at 4 d postdose. Pearson's correlation coefficient (*r*) with significance level (*P*) and equation for the linear relation are displayed.

**FIGURE 3:**
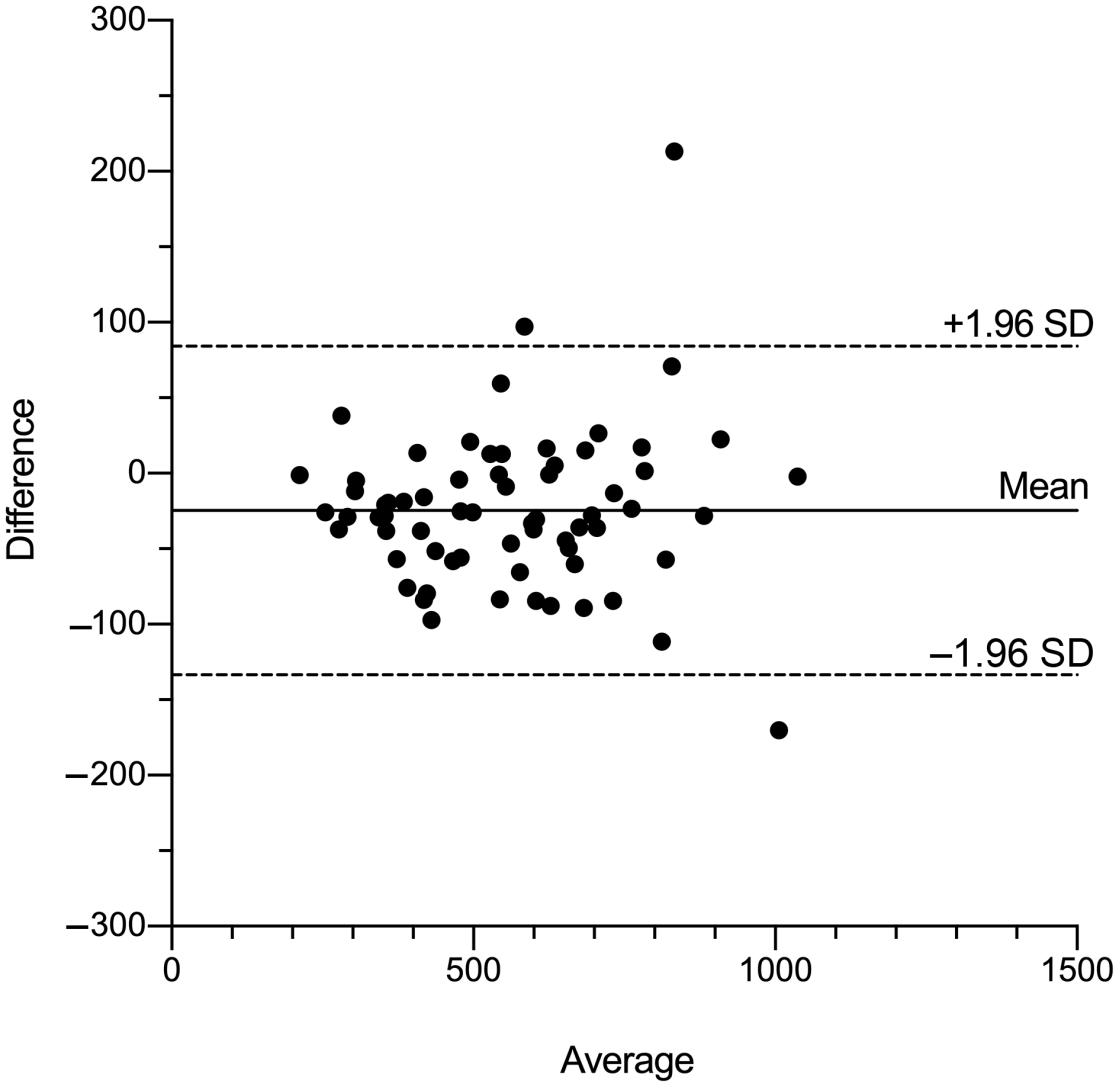
Bland–Altman comparison of total body store (TBS) determinations from dried serum spots (DSS) and liquid plasma (LP) paired samples (*n* = 72) at 4 d postdose. Solid line shows the mean difference with dashed lines displaying 95% limits of agreement (± 1.96 SD).

Mean TBS estimates in Filipino children were 555 μmol (± 191 SD) from DSS and 530 μmol (± 188 SD) from liquid plasma (Table 2). As the TBS 4-d RID equation is largely driven by the stable isotope tracer to tracee ratio, SAp from plasma was plotted against the SAp from DSS (**Figure 2**) which yielded a Pearson's correlation coefficient (*r*) of 0.945 (*P* < 0.01). Good agreement of TBS determinations between the 2 sampling methods was confirmed by a Bland–Altman plot (**Figure 3**) with an intraindividual difference of 24.7 μmol (4.6%). From TBS determinations in DSS and plasma, calculated TLR mean values were 1.7 μmol/g (± 0.6 SD) and 1.6 μmol/g (± 0.6 SD), respectively. Comparison of total retinol ([^12^C]+[^13^C_10_]) concentrations between DSS and plasma samples resulted in respective mean values of 1.05 μmol/L and 1.13 μmol/L.

## Discussion

In RID studies, the size of the administered VA tracer dose, the sampling time point for TBS determination, and the volume of blood required are dependent on the sensitivity of the MS method available. Furthermore, both the volume and frequency of blood draws are limited when carrying out VA assessments in infants and young children. In the present study, 100% of subject samples were above the LC-MS/MS tracer LOQ when 3 DSS (120 μL of applied serum) were analyzed from 4 d time points postdose. To our knowledge, this study confirms, for the first time, that TBS determinations from DSS are comparable to standard plasma samples, thus making DSS an attractive alternative method for population-based VA status assessments without the need for dry ice shipment. Although previous RID studies have also administered a VA stable isotope dose to children around the RDA, a substantially larger volume of frozen serum (1.0–1.5 mL) was required for analysis by GC-combustion-isotope ratio MS (GC-C-IRMS) (6, 7, 32–35), thus prohibiting the use of DSS using this technology. Furthermore, VA tracer analysis by GC-C-IRMS is more time-consuming requiring prior sample HPLC purification, derivatization, and additional baseline blood samples to ascertain the natural abundance of [^13^C]. Although the LOD and LOQ of [^13^C_10_]retinol presented here is a modest improvement on our previously reported LC-MS/MS method (27), optimization of the sample extract resuspension solvent composition was critical for detection of the tracer from DSS. This allowed for a 5-fold greater sample injection volume without compromising peak shape or baseline resolution.

Apart from the size of administered VA tracer dose, the time point at which blood samples are drawn for TBS determinations has an impact on the ability to detect the tracer from a limited volume of serum applied as DSS. Reductions in circulating tracer occur due to exchange with small and large extravascular body pools and irreversible catabolic losses during the SAp terminal slope (22). Although adequate time is required for the tracer to equilibrate with extravascular body pools postdose, samples must be drawn before the analytical LOQ is reached. Previously, TBS determinations utilizing RID have typically ranged between 3 d and 26 d in a variety of age groups and population settings (5). However, it has been demonstrated that the optimal sampling time for TBS determination is 4–5 d postdose when a modified RID equation was applied to healthy young adults in the UK (28). More recently, using the same equation in combination with a “super-child” approach, it was found that there was no significant difference in TBS predictions at 4 d compared with later time points ≤21 d postdose (29). Advantages of earlier sampling time points are greater subject study compliance with decreased changes in health status, such as inflammation, which can affect VA kinetics. Although there are no current cut-offs for VA status utilizing TBS, derived TLR values have been proposed for classifying individuals with hypervitaminosis A at concentrations >1.0 μmol/g and VA toxicity at >10 μmol/g (8). In this respect, the Philippine cohort under study here would fall into the hypervitaminotic category, however current VA status cut-offs may require revision (36).

Whatman 903 filter paper cards are convenient blood microsampling devices which not only reduce the reliance on a cold chain for shipping to analytical laboratories, but also reduce the risk of disease transmission from contaminated samples (37). Although sampling of capillary blood is minimally invasive, analysis of retinol from DBS is technically more challenging compared with DSS. For example, direct application of whole blood is subject to hematocrit bias and differential spreading/saturation across the paper (38). Thus, an analytical correction factor has to be applied to relate punched DBS to serum retinol concentration such as an additional analysis of sodium concentration (16) or hematocrit (17). Furthermore, conflicting reports remain over retinol stability in DBS. Craft et al. (15) demonstrated that retinol degraded over the first 6–10 d at all 3 storage temperatures (−20°C, 4°C, 25°C) before stabilizing. Similarly, Erhardt et al. (16) reported an 18–23% decrease in retinol concentration during 1 wk of storage at room temperature with retinol remaining stable for >3 mo thereafter. Conversely, Fallah et al. (17) found that retinol was stable in DBS for 1 wk at room temperature and for a further 3 mo at −20°C, whereas Huang et al. (18) reported continued stability over 33 d at room temperature. Interestingly, both DBS extraction solutions contained a weak organic acid which facilitates the dissociation of retinol from RBP in the DBS matrix (18). In the present study, there was no degradation of retinol in DSS when stored frozen then shipped at ambient temperature which significantly reduced shipping costs. Although DSS require a venous blood draw and centrifugation, the advent of portable battery-operated centrifuges could also make the RID technique more accessible in community settings.

In summary, this is the first study to confirm that TBS can be determined from DSS using the RID technique and a physiological VA tracer dose. The single blood draw at 4 d postdose and small volume of serum applied as DSS makes the methodology especially applicable for VA status assessments in young children. Further validation, in terms of retinol stability, is required to establish whether the cold chain can be eliminated entirely during the storage of DSS for LC-MS/MS analysis.

## Data Availability

All datasets of the GloVitAS project funded by the Bill and Melinda Gates Foundation (OPP1115464: Assessing the risk of vitamin A toxicity due to large scale food fortification and other interventions) will be made publicly available after publication of all available results, but latest 2 years after the end of the project. Supporting data to publications will further be made openly available via the Newcastle University data depository.

## Notes

Support for this work was provided by the Bill & Melinda Gates Foundation (Project Number: OPP1115464), the International Atomic Energy Agency (IAEA) in Vienna (Project Number E4.30.30), and the Medical Research Council (MRC, UK) Confidence in Concept grant (MC_PC_16054). Data supporting this publication are openly available at https://doi.org/10.25405/data.ncl.9771902.v1.

Author disclosures: The authors report no conflicts of interest.

Abbreviations used: AGP, α(1)-acid glycoprotein; CRP, C-reactive protein; DBS, dried blood spots; DSS, dried serum spots; GC-C-IRMS, GC-combustion-isotope ratio MS; GloVitAS, Global vitamin A safety assessment; IS, internal standard; LMIC, low- and middle-income countries; LOQ, limit of quantitation; LP, liquid plasma; NIST, National Institute of Standards and Technology; OFN, oxygen-free nitrogen; QC, quality control; RBP, retinol-binding protein; RID, retinol isotope dilution; SAp, specific activity in plasma/serum; SRM, selected reaction monitoring; TBS, total body stores; TLR, total liver reserves; VA, vitamin A; VAD, vitamin A deficiency; VAS, vitamin A supplement.
